# Effects of Regulating Positive Emotions through Reappraisal and Suppression on Verbal and Non-Verbal Recognition Memory

**DOI:** 10.1371/journal.pone.0062750

**Published:** 2013-04-26

**Authors:** Catherine N. M. Ortner, Monica de Koning

**Affiliations:** Thompson Rivers University, Kamloops, British Columbia, Canada; University of Groningen, The Netherlands

## Abstract

Previous research has suggested that regulating emotions through reappraisal does not incur cognitive costs. However, in those experiments, cognitive costs were often assessed by recognition memory for information that was contextually related to the emotionally evocative stimuli and may have been incorporated into the reappraisal script, facilitating memory. Furthermore, there is little research on the cognitive correlates of regulating positive emotions. In the current experiment, we tested memory for information that was contextually unrelated to the emotional stimuli and could not easily be related to the reappraisal. Participants viewed neutral and mildly positive slides and either reappraised, suppressed their emotions, or viewed the images with no emotion regulation instruction. At the same time, they heard abstract words that were unrelated to the picture stimuli. Subsequent verbal recognition memory was lower after reappraising than viewing, whereas non-verbal recognition memory (of the slides) was higher after reappraising, but only for positive pictures and when participants viewed the positive pictures first. Suppression had no significant effect on either verbal or non-verbal recognition scores, although there was a trend towards poorer recognition of verbal information. The findings support the notion that reappraisal is effortful and draws on limited cognitive resources, causing decrements in performance in a concurrent memory task.

## Introduction

Most studies of emotion regulation to date have examined the down regulation of negative emotional responses [1], [2], [3]. However, individuals may also modulate positive emotions using reappraisal or suppression. For example, when seeing a co-worker slip on a wet floor in front of a ‘wet floor’ sign at work, an employee may feel amused at the situation but in consideration of their co-worker’s feelings may reinterpret the situation (reappraisal) by thinking about the potential negative consequences of the situation (perhaps the co-worker is injured) or suppress their emotional expressions of amusement. In addition, some forms of psychopathology are characterised not only by dysregulation of negative affect but also of positive affect [4]. For example, depression is posited to involve a dysregulation of reward processing, resulting in lower positive affect [5], [6], and research has shown that the tendency to experience positive affect in response to positive events is independent from the tendency to experience negative affect in response to negative events [6]. Therefore, it is important to examine the regulation of positive affective responses and how this may differ from the regulation of negative affect. Studies to date have suggested that down-regulating the response to positive emotional stimuli results in reductions in the subjective and physiological response [7], as well as a reduction in the late positive component of the event-related potential, thought to reflect attentional salience [8].

The effects of emotion regulation on cognition are less clear cut and researchers to date have not examined the cognitive costs of reappraising positive events. Research has shown that engaging in suppression while viewing negative slides reduces verbal memory for information that was paired with those slides [9], [10], but reappraisal of negative emotions has no effect on verbal memory and actually enhances non-verbal memory (i.e., memory for the slides themselves) compared to a no emotion regulation condition [9], [11], [12]. These studies have been taken as evidence that reappraisal is an antecedent-focused emotion regulation strategy–occurring early in the development of an emotional response–and that therefore it is not cognitively costly. An alternative explanation is that emotional stimulus encoding during reappraisal is facilitated due to elaboration, resulting in better subsequent recognition of that stimulus [11]. Furthermore, verbal information that is contextually related to the emotionally evocative stimuli [10] may be attended to and therefore encoded, facilitating later recognition. In contrast, suppression requires the participant to direct attention internally–to their own facial expressions of emotion. This should reduce encoding of information and produce subsequent poorer recall.

Reappraisal of both negative and positive events is a dynamic process that unfolds over time and recruits brain regions involved in working memory, response inhibition, selective attention, and monitoring, in order to select, implement, maintain, and update a reappraisal strategy [12], [13], [14]. Furthermore, higher working memory capacity is associated with more effective reappraisal of negative emotional stimuli [15], [16]. However, the working memory requirements of reappraising positive emotional stimuli have not yet been examined.

Cognitive load theory suggests that, when performing two cognitive tasks simultaneously, performance on one or both tasks suffers if those tasks are challenging enough (i.e., high in cognitive load) [17]. Thus, if reappraisal of positive and negative stimuli is cognitively demanding, through its working memory, inhibition, selective attention, and monitoring requirements [14], it should reduce the resources available for concurrent task performance, when that task is irrelevant to the emotionally evocative stimuli. This has been demonstrated in a study examining the effect of reappraisal of negative stimuli on concurrent reaction time performance–participants were slower to respond on a concurrent, simple reaction time task when reappraising negative images, compared to when passively viewing them [18]. However, there is a gap in the literature regarding the cognitive correlates of reappraisal when viewing positively valenced stimuli.

In the current study, we sought to examine the effects of reappraisal and suppression during positive images on verbal and non-verbal recognition. Participants viewed mildly positive emotionally evocative images and either reappraised, suppressed their expressions, or viewed the pictures with no emotion regulation instructions (control condition). At the same time, abstract words were presented auditorily. Later, participants completed recognition tasks of the abstract words (verbal memory) and the pictures (non-verbal memory). We expected that presenting participants with verbal information that was *unrelated* to the emotionally evocative stimuli (i.e., abstract words), and therefore not readily incorporated into the reappraisal script, would mitigate facilitation of encoding of the information into memory. Furthermore, we expected that reappraisal would constitute a cognitive load, reducing encoding of verbal information into memory. For both reappraisal and suppression, encoding of unrelated verbal information should be reduced, compared to a no emotion regulation condition. Encoding of non-verbal information (the emotional stimuli) should remain intact or be enhanced during reappraisal, because attention is focused on the emotional stimulus in order to generate a reappraisal–stimulus elaboration may facilitate encoding. Suppression should also reduce encoding of non-verbal information [10], because attention needs to be directed away from the emotionally evocative stimulus in order to maintain and monitor one’s facial expressions.

## Methods

### Participants

Participants were 65 undergraduate students from the Thompson Rivers University participant pool.

### Ethics Statement

Ethical approval was granted from the Thompson Rivers University Psychology Department Research Ethics Committee – Human Subjects. All participants gave written informed consent and received 2% course bonus credit for their participation.

### Stimuli

#### Affective picture stimuli

Stimuli were nine neutral and nine mildly positively valenced pictures from the International Affective Picture System (IAPS) [19]. The IAPS manual provides normative ratings of valence (1 = most unpleasant to 9 = most pleasant) and arousal (1 = least aroused to 9 = most aroused) for all the images, from approximately 100 college students [19]. We did not include unpleasant images (as used by Richards & Gross) [10], because the experiment was part of a student project. In order to gain ethics approval in a timely manner, the project was required to be minimal risk and therefore we were unable to include unpleasant images. Mildly positive pictures were selected to have valence ratings greater than 6 (mean valence = 7.31; range = 6.88 to 7.68) and neutral pictures were selected to have valence ratings less than 6 (mean valence = 4.80; range = 3.65 to 5.78). Actual IAPS picture numbers were as follows: neutral images, 1390, 7037, 7100, 7560, 9210, 2191, 7640, 2039, 7620; positive images, 2398, 2501, 5621, 5629, 5831, 8490, 2655, 2388, 2091. The positive images depicted, for example, people skydiving or children playing. The neutral pictures depicted, for example, a man working in a field or a fire hydrant. The stimuli included in the non-verbal recognition task consisted of the original pictures and three lures that were variations of each target picture that had been modified using picture editing software: one lure with the colour saturation changed, one lure that was a mirror image of the original stimulus, and one lure with both modifications.

#### Verbal stimuli

Verbal stimuli were eighteen abstract nouns, selected from the MRC psycholinguistic database to be low in concreteness and more than four letters long [20] (examples include “extra,” “concept,” and “dimension”). The spoken words were recorded so that they could be played over headphones during picture viewing. The stimuli included in the non-verbal recognition task consisted of the original stimuli and three lures that were synonyms of the target word.

### Procedure and Design

The experimenter tested participants in small groups in sessions lasting approximately 40 minutes, with each participant sitting at a computer where the picture presentation task was to be presented. Participants were randomly assigned to one of three emotion regulation conditions–reappraisal to upregulate feelings of negative emotion, suppression of emotional expression, or view (no emotion regulation), as they viewed neutral and mildly positive images.

#### Picture viewing instructions

In the View condition, participants received no emotion regulation instruction but were told to:

“Please view the slides as you normally would.”

In the reappraisal condition, participants received reappraisal instructions adapted from Richards and Gross [10]. Because the images employed in the current study were neutral or only mildly positive, we asked participants to regulate their emotions by thinking about a negative outcome:

“Please view the slides carefully when they are presented to you. In addition, I would like to see how well you can control the way you view things. Therefore, it is important that you try your best to adopt a negative attitude while viewing the slides. In other words, as you view the slides, try to think about them in as negative a way as possible. Try to make up an unpleasant story for each picture you are shown.”

For example, when viewing a picture of a child playing on the beach, a participant could imagine that a rogue wave is about to sweep the child away.

In the suppression condition, participants received the following instructions, adapted from Richards and Gross [10]:

“Please view the slides carefully, when they are presented to you. In addition, I would like to see how well you can control your facial expressions. Therefore, it is important that you try your best to adopt a neutral facial expression as you watch the slides. To do this, please try to keep a straight face by keeping the muscles around your neck, chin, lips, cheeks, eyes and forehead very still. So watch the slides carefully, but please try to keep your facial muscles still so you do not make any expressions at all.”

Comprehension of instructions was ensured during practice trials.

#### Picture presentation task

We used E-Prime software to show participants the pictures in two runs of nine (neutral and mildly positive images) presented in counterbalanced order across participants. Each image appeared on the screen for 10 s (following Richards & Gross) [10], with the abstract word presented at 3 s after picture onset. Pairing of abstract words with picture stimuli was determined randomly for each participant. After the first run of nine images, participants rated the extent to which they felt six emotions (sadness, anger, distress, happiness, enthusiastic, interested) on a seven-point Likert-type scale (0 = not at all, 6 = very much). Participants were then reminded of the emotion regulation instructions before they completed the second run of nine images and made the subjective ratings a second time. Participants then worked on a paper and pencil maze task [21] for three minutes as a distractor task. Finally, they completed the non-verbal and verbal memory tasks. For both tasks, participants were required to be as accurate as possible, but there was no time limit. For the non-verbal memory task, participants viewed each original stimulus with the three picture lures arranged in random positions in a spread. Participants selected the picture they thought most closely resembled the picture they had seen in the picture viewing task. For the verbal memory task, participants were required to identify each target word when it was presented in a list with the three lures for that target word. Again, the target word and lures were presented in random order. Both the verbal and non-verbal memory tests were intended to be measures of incidental memory: at no time did we instruct participants to try to remember either the pictures or the words.

Finally, participants completed the Emotion Regulation Questionnaire [2] to assess individual differences in tendencies to use reappraisal and suppression (the data from the Emotion Regulation Questionnaire form part of another study and will not be presented here).

## Results

### Data Analyses

We computed mean positive (happiness, enthusiastic, interested) and negative (sadness, anger, distress) affect scores for each participant. Verbal and non-verbal memory scores were computed by totalling the number of correct responses for each picture valence (neutral and positive) separately. Preliminary analyses indicated that picture presentation order (neutral pictures first or positive pictures first) significantly accounted for some of the variance in memory scores and subjective ratings. Therefore, order was included as a factor in the analyses, although the effect of order will not be discussed at length as it is beyond the scope of the current experiment. Random assignment of participants resulted in *n* = 11 in the view, neutral first condition; *n* = 10 in the view, positive first condition; *n* = 11 in the reappraise, neutral first condition; *n* = 11 in the reappraise, positive first condition; *n* = 10 in the suppress, neutral first condition; and *n* = 12 in the suppress, positive first condition. Subjective ratings data were missing for two participants (one from the reappraise, neutral first condition, and one from the view, positive first condition).

### Subjective Ratings

As a manipulation check, we investigated the effect of Strategy (reappraise, suppress, view), Picture Valence (neutral, positive), and Order (neutral images first, positive images first) on positive and negative affect ratings, with two 3×2×2 mixed ANOVAs. For positive affect, there was a significant effect of Strategy, a significant effect of Picture Valence, and a significant interaction between Order and Strategy, *F*(2, 57) = 4.15, *p* = .02, *η_G_^2^* = .12, *F*(1, 57) = 13.17, *p*<.001, *η_G_^2^* = .02, and *F*(2, 57) = 4.81, *p* = .01, *η_G_^2^* = .13, respectively. No other interactions were significant (see Table 1 for means and standard deviations). Participants gave higher ratings of positive affect when viewing positive than neutral pictures. In order to explore the effect of Strategy and the interaction between Order and Strategy, four *t*-tests were conducted, comparing reappraise with view and suppress with view, for each order (neutral images first and positive images first) separately. We applied the Bonferroni correction for multiple comparisons (α = 0.05/4 = .0125). Only the difference between suppress and view was significant, when neutral images were presented first, *t*(18.76) = 3.21, *p* = .002, participants giving lower positive affect ratings for suppress than view.

**Table 1:** Mean Positive Affect Ratings by Strategy (Reappraise, Suppress, or View), Picture Valence (Neutral or Positive), and Order (Neutral or Positive Pictures first).

	Order			
	Neutral Pictures First		Positive Pictures First	
	Picture Valence			
Strategy	Neutral	Positive	Neutral	Positive
	Rating (0–6)			
View				
*M*	3.18	3.79	2.67	3.15
*SD*	1.16	1.05	1.38	1.73
Reappraise				
*M*	2.60	2.50	1.73	2.12
*SD*	0.84	0.82	1.03	0.91
Suppress				
*M*	1.90	2.13	3.11	3.39
*SD*	1.18	1.06	1.32	1.31

For negative affect, there was a significant effect of Strategy on negative affect ratings, *F*(2, 57) = 10.85, *p*<.001, *η_G_^2^* = .24, but no effect of Order or Picture Valence, and no interactions (see Table 2 for means and standard deviations). Further tests (collapsing across Order and Picture Valence, and adjusting for multiple comparisons, α = .05/2 = .025) indicated higher negative affect ratings for reappraise than view, *t*(32.81) = 3.43, *p* = .002, but no significant difference between suppress and view *t*(39.30) = 0.4408, *n.s.*

**Table 2:** Mean Negative Affect Ratings by Strategy (Reappraise, Suppress, or View), Picture Valence (Neutral or Positive), and Order (Neutral or Positive Pictures first).

	Order			
	Neutral Pictures First		Positive Pictures First	
	Picture Valence			
Strategy	Neutral	Positive	Neutral	Positive
	Rating (0–6)			
View				
*M*	0.88	0.88	0.81	0.70
*SD*	0.92	1.13	0.97	0.99
Reappraise				
*M*	1.53	1.47	2.33	2.52
*SD*	1.31	1.07	1.49	1.25
Suppress				
*M*	0.60	0.53	0.94	0.75
*SD*	0.54	0.69	1.20	0.81

### Verbal Memory

**Figure 1:**
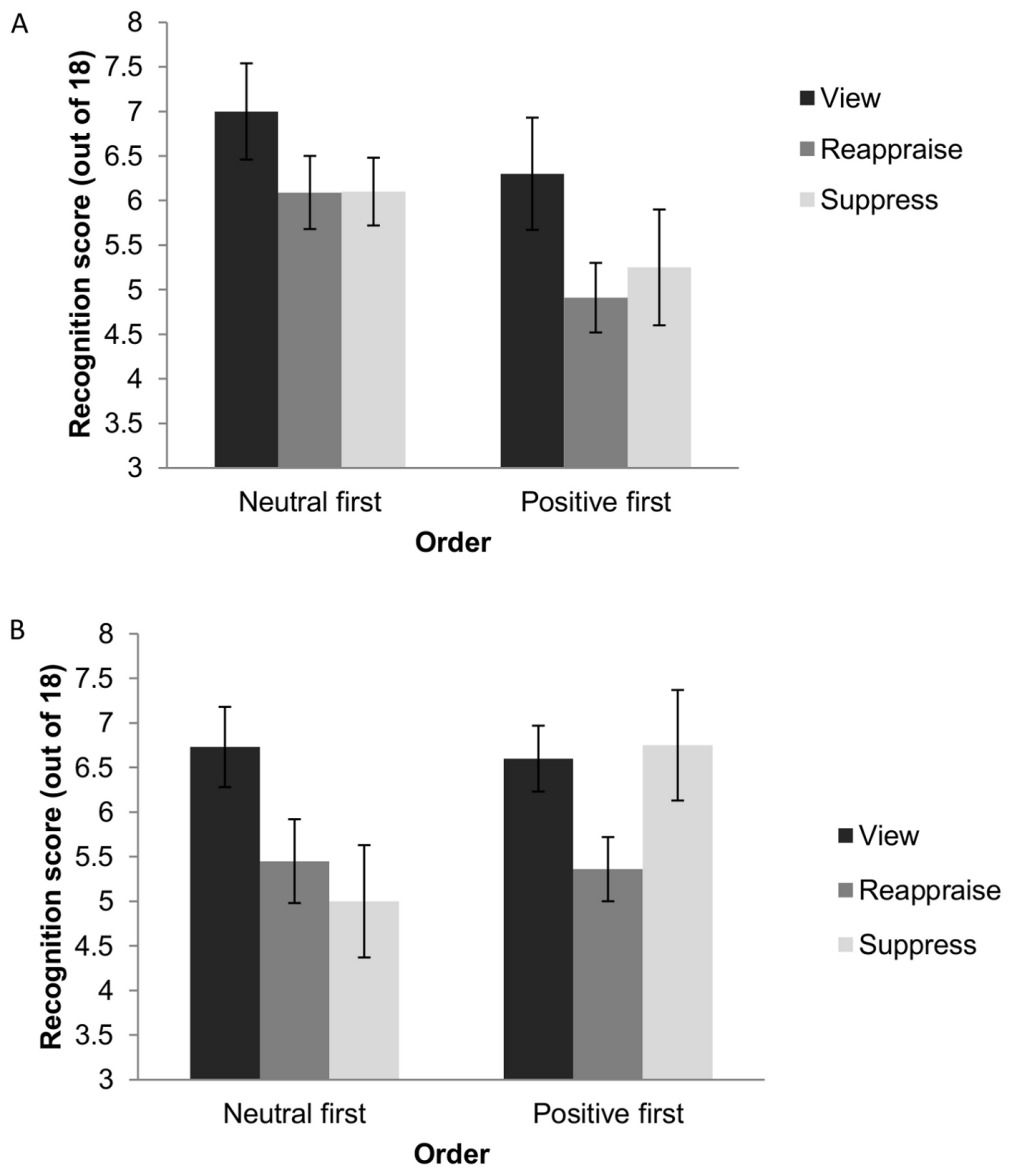
Verbal memory scores for neutral (A) and positive (B) pictures. Legend: Verbal memory score according to emotion regulation strategy (reappraise, suppress, or view)and order (neutral or positive pictures first); error bars represent standard error of the mean.

We conducted a 3×2×2 mixed ANOVA to examine the effects of Strategy (reappraise, suppress, view), Picture Valence (neutral, positive), and Order (neutral images first, positive images first) on verbal memory scores. There was a main effect of Strategy and an interaction between Order and Picture Valence, *F*(2, 59) = 3.85, *p* = .026, *η_G_^2^* = .09, and *F*(1, 59) = 12.13, *p*<.001, *η_G_^2^* = .047, respectively. To follow up on the main effect of Strategy and to test the hypothesis that reappraisal and suppression would reduce verbal memory compared to the view condition, we conducted planned comparisons (collapsing across Order and Picture Valence, and correcting for multiple comparisons, α = .05/2 = .025). Participants in the reappraise condition recognised significantly fewer words than participants in the view condition, *t*(39.33) = 3.07, *p* = .002. Participants in the suppress condition recognised fewer words than participants in the view condition, although this difference only approached significance, *t*(39.92) = 1.83, *p* = .037 (see Figures 1A and 1B).

To take into account the possibility that negative affect, and not just the act of regulating one’s emotions, might also influence encoding of information into memory, we conducted a one-way Analysis of Covariance to examine the effects of Strategy (reappraise, suppress, view) on verbal memory scores, while controlling for negative affect, collapsing across Order and Picture Valence. There remained a significant effect of Strategy on verbal memory, *F*(2, 59) = 3.24, *p* = .046, and there was no effect of the covariate, negative affect, on verbal memory, *F*(1, 59) = 1.57, *n.s.*

### Non-verbal Memory

**Figure 2:**
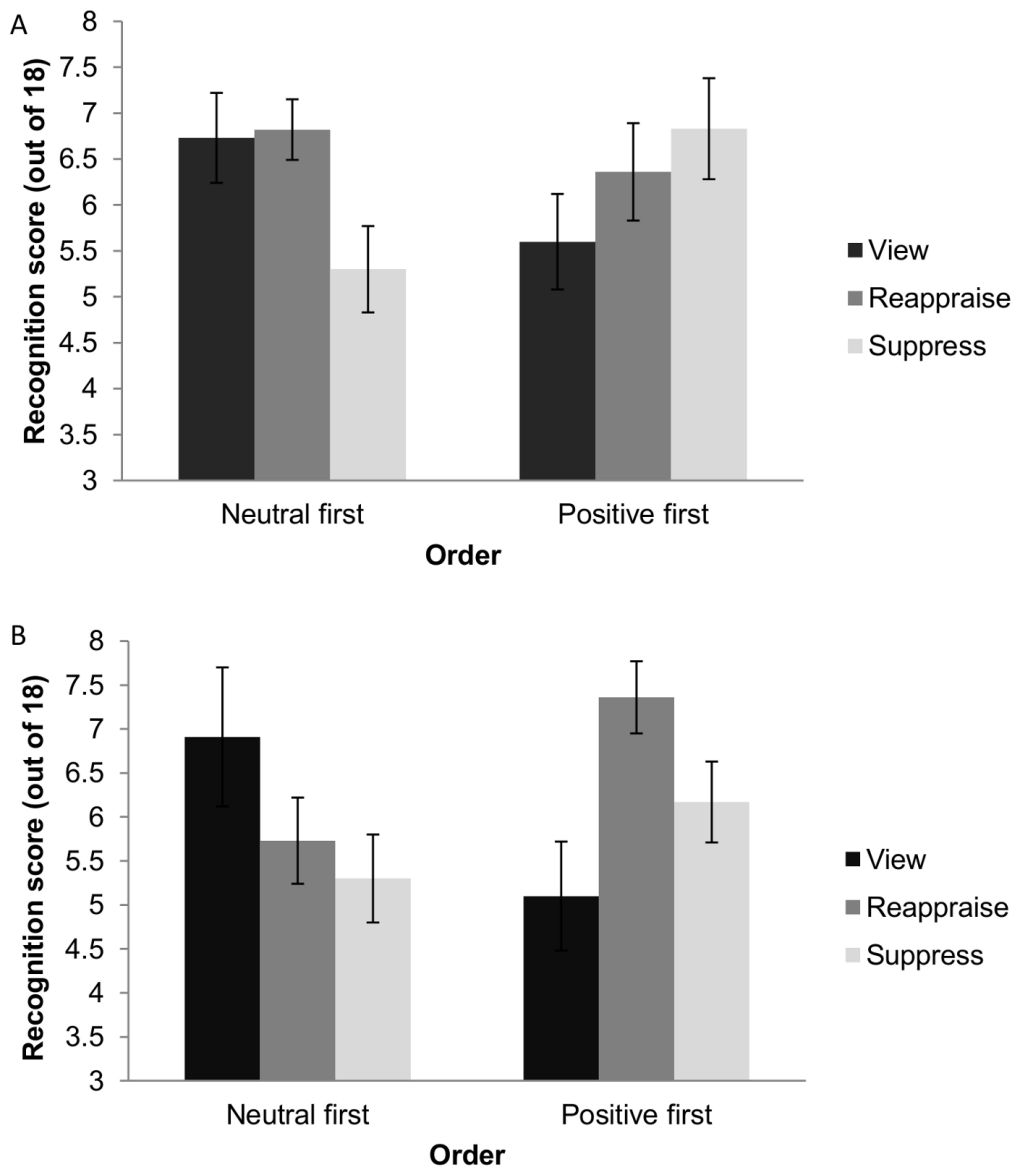
Non-verbal memory scores for neutral (A) and positive (B) pictures. Legend: Non-verbal memory score according to emotion regulation strategy (reappraise, suppress, or view)and order (neutral or positive pictures first); error bars represent standard error of the mean.

We conducted a 3×2×2 mixed ANOVA to examine the effects of Strategy (reappraise, suppress, view), Picture Valence (neutral, positive), and Order (neutral images first, positive images first) on non-verbal memory scores. There was an interaction between Strategy and Order, and a three-way interaction between Strategy, Order, and Picture Valence, *F*(2, 59) = 4.34, *p* = .018, *η_G_^2^* = .11, and *F*(2, 59) = 6.04, *p* = .004, *η_G_^2^* = .04, respectively. To decompose the three-way interaction, eight *t*-tests were conducted, comparing non-verbal memory scores for reappraise and view and suppress and view, for positive and neutral pictures, and for each order, separately. We corrected for multiple comparisons, α = .05/8 = .006. Participants in the reappraise condition showed significantly higher non-verbal memory scores than participants in the view condition when reappraising positive pictures *and* when positive images were presented first *t*(15.83) = 3.03, *p* = .004. In no other conditions was there a significant difference between reappraise and view, and there were no differences in non-verbal memory scores between suppress and view (see Figures 2A and 2B).

## Discussion

The current experiment assessed the effect of reappraisal and suppression on recognition memory for neutral and mildly positive pictures (non-verbal memory) and for abstract words heard during the picture presentation (verbal memory). Based on neuroimaging evidence [14], cognitive load theory [17], and recent evidence of the cognitive costs of reappraisal [18], we predicted that reappraisal would reduce verbal memory and increase non-verbal memory. We also predicted that suppression would reduce non-verbal memory. Our findings partially confirmed these predictions. Participants who reappraised the pictures showed lower verbal recognition scores than participants in the view condition. Reappraisal was also associated with higher non-verbal memory scores, but only for positive pictures and only in participants who viewed the positive pictures before the neutral images. There was no effect of suppression on either verbal or non-verbal memory, but a trend towards poorer verbal memory than when viewing. The findings support the notion that reappraisal of positive stimuli constitutes a cognitive load–that is, it draws on a limited capacity of cognitive resources, causing decrements in concurrent task performance.

These results may appear to contradict earlier findings by Richards and Gross [10], who showed that reappraisal had no effect on verbal memory. However, they can be explained by considering the differences in the verbal memory tasks in the two studies. In Richards and Gross’ experiment, the verbal information presented concurrently with the images was contextually related to the emotionally-evocative slides (i.e., the name, occupation, and cause of injury in the individual in the picture) and may have been attended to and incorporated into the reappraisal script, thus facilitating encoding into memory. As such, this may not be a sufficient test of the cognitive costs of reappraisal. In the current study, we deliberately selected verbal targets that were unrelated to the neutral and positive pictures and were abstract, so that they could not easily be incorporated into a reappraisal script. In this case, we were able to demonstrate significant decrements in verbal memory performance in the reappraisal condition.

We did not find a consistent effect of reappraisal on non-verbal recognition scores. Other researchers [11] have found that free recall of unpleasant images is improved after either enhancing or decreasing negative emotion in response to those images. One explanation for this discrepancy is that prior research [11] used a slightly different reappraisal instruction where participants were instructed to either increase or decrease the personal relevance of the slides. This may have resulted in more effective stimulus elaboration, and therefore greater facilitation of encoding, than our more general instruction to “try to think about the slides in a more negative way.”

### Limitations and Future Directions

An alternative explanation for our finding of a decrement in verbal memory performance in the reappraisal condition is that it was not reappraisal per se that reduced verbal memory, but the increase in negative affect brought about by upregulating negative emotional experience in response to the slides. There has been little research on the cognitive costs of reappraisal of positive and neutral stimuli. Based on Kalisch’s [14] analysis of the components of reappraisal, we predicted that reappraising positive stimuli should involve the same processes of strategy selection, implementation, maintenance, and updating as reappraisal of unpleasant stimuli. However, it is possible that by reappraising mildly positive and neutral stimuli, participants felt increased negative emotion and that this negative emotion affected encoding of verbal information into long-term memory. Indeed, other research has suggested that experiencing feelings is effortful and draws on cognitive resources [22]. Given that we found that reappraisal increased subjective ratings of negative affect, this explanation seems plausible. However, we tested this interpretation by conducting further analyses where we controlled for negative affect when analysing the effect of emotion regulation strategy on verbal memory. Negative affect was not related to verbal memory scores and the effect of emotion regulation strategy on verbal memory remained significant even when controlling for negative affect. Findings by Dillon et al. [11], where both up- and down-regulation of emotion enhanced free recall, also suggest that changes in arousal as a result of reappraisal do not influence encoding. To fully rule out this explanation in the current design, further research should replicate the experiment using unpleasant images and reappraisal to down-regulate negative emotion experience. It is notable, however, that the current demonstration of the cognitive load of reappraisal concurs with recent research showing attentional costs when reappraising unpleasant stimuli [18].

Another consideration is that generating and maintaining a reappraisal script may tax verbal working memory processes in particular. Based on the view that working memory resources are domain-specific [23], one should therefore expect that encoding of verbal information that is not related to the reappraisal task would be reduced. Further research could use non-verbal stimuli (which are unrelated to the emotionally evocative stimuli) for later recognition or recall to test whether reappraisal also impinges on non-verbal encoding.

We found no effect of suppression on either verbal or non-verbal memory, although there was a trend towards reduced verbal memory. The discrepancy between this finding and Richards and Gross’ [9], [10] findings of reduced non-verbal memory as a result of reappraisal may be explained by our use of neutral and mildly positive images. It is likely that these stimuli evoked only a weak emotional response. As a result, the cognitive effort required to suppress facial expressions of emotion would have been minimal, leaving sufficient resources for verbal and non-verbal encoding. Replicating this study with unpleasant images should increase the cognitive load of suppression, yielding the same findings of reduced non-verbal memory as Richards and Gross [10].

Finally, given the potential clinical implications of these findings for individuals undergoing cognitive therapy for depression, it would be interesting to explore the cognitive load of reappraisal in depressed clients using a similar task.

### Conclusion

In sum, the current experiment demonstrates that, when presented with verbal information that is contextually unrelated to mildly emotionally evocative stimuli, reappraisal to increase negative emotion bears cognitive costs, and that these costs appear to be unrelated to the influence of negative feelings on cognition.
